# Fgf signaling controls the telencephalic distribution of Fgf-expressing progenitors generated in the rostral patterning center

**DOI:** 10.1186/s13064-015-0037-7

**Published:** 2015-03-31

**Authors:** Renée V Hoch, Jeffrey A Clarke, John LR Rubenstein

**Affiliations:** Department of Psychiatry, University of California, 1550 4th Street, UCSF MC 2611, San Francisco, CA 94158 USA; Current address: PLOS, 1160 Battery Street, San Francisco, CA 94111 USA

**Keywords:** Fgf8, Fgf17, Telencephalon, Development, Septum, Lineage, Fate map, Progenitors, Cholinergic, MGE

## Abstract

**Background:**

The rostral patterning center (RPC) secretes multiple fibroblast growth factors (Fgfs) essential for telencephalon growth and patterning. Fgf expression patterns suggest that they mark functionally distinct RPC subdomains. We generated *Fgf8*^*CreER*^ and *Fgf17*^*CreER*^ mice and used them to analyze the lineages of Fgf8- *versus* Fgf17-expressing RPC cells.

**Results:**

Both lineages contributed to medial structures of the rostroventral telencephalon structures including the septum and medial prefrontral cortex. In addition, RPC-derived progenitors were observed in other regions of the early telencephalic neuroepithelium and generated neurons in the olfactory bulb, neocortex, and basal ganglia. Surprisingly, Fgf8^+^ RPC progenitors generated the majority of basal ganglia cholinergic neurons. Compared to the Fgf8 lineage, the Fgf17 lineage was more restricted in its early dispersion and its contributions to the telencephalon. Mutant studies suggested that Fgf8 and Fgf17 restrict spread of RPC progenitor subpopulations.

**Conclusions:**

We identified the RPC as an important source of progenitors that contribute broadly to the telencephalon and found that two molecularly distinct progenitor subtypes in the RPC make different contributions to the developing forebrain.

**Electronic supplementary material:**

The online version of this article (doi:10.1186/s13064-015-0037-7) contains supplementary material, which is available to authorized users.

## Background

In the developing telencephalon, the rostral patterning center (RPC) secretes multiple fibroblast growth factors (Fgf3, Fgf8, Fgf15, Fgf17, Fgf18) that are essential for regional patterning, proliferation, and cell survival (reviewed in [1]). This signaling center in the rostromedial neuroepithelium is active from neural plate stages through mid-embryogenesis. Genetic and experimental embryological analyses have demonstrated that Fgf8 and Fgf17 serve different roles in the RPC. Fgf8 impacts proliferation and cell survival in the early rostral telencephalon, affects patterning of the cerebral cortex and basal ganglia, and is required for the formation of olfactory bulbs (OBs) and midline structures [2-11]. In contrast, Fgf17 is required only for select roles in rostral-caudal cortical patterning and development of the dorsomedial prefrontal cortex (PFC; [12,13]). The mechanisms by which Fgf8 and Fgf17 exert their distinct developmental functions are not yet clear.

Several biochemical mechanisms may contribute to functional distinctions between RPC Fgfs. Fgf8 protein has a graded distribution in the telencephalon and has been reported to act as a morphogen to drive cellular responses distal to the RPC [14]. Fgf17 protein distribution has not been described, but it is possible that Fgf8 and Fgf17 have different diffusion properties - and thus protein distributions - in the early telencephalon. In addition, Fgf8 and Fgf17 may activate distinct receptors and/or signaling pathways, thereby inducing different cellular responses in the early telencephalon. In support of this, Fgf8 and Fgf17 exhibit different receptor binding affinities in surface plasmon resonance studies [15], and brain explant studies revealed that Fgf17b and Fgf8b isoforms differentially regulate gene expression [16]. These mechanisms could enable the related ligands to target different cell populations and/or induce distinct responses *in vivo*.

Fgfs are expressed in distinct subdomains of the late neurula stage RPC: *Fgf8* is expressed in a medial domain nested within (possibly excluded by) broader *Fgf15*^*+*^ and *Fgf17*^*+*^ domains [17,13,18]. We hypothesized that Fgfs mark functionally distinct RPC subdomains, and that fate maps of *Fgf8*^+^*versus Fgf17*^+^ RPC cells would provide novel insight into mechanisms underlying their functional diversification. Previous dye injection and tissue graft studies demonstrated that the rostromedial RPC (anterior neural ridge, ANR) gives rise to the commissural plate, septum, and select rostral components of the basal ganglia and cortex [19-22]. While these experiments provided a broad overview of RPC derivatives, their methodologies did not allow analysis of molecularly distinct RPC subpopulations. Therefore, we generated *Fgf8*^*CreER*^ and *Fgf17*^*CreER*^ knock in mice and conducted comparative lineage analyses. We found that the Fgf8 and Fgf17 RPC lineages differentially contribute to the mature telencephalon. *Fgf8*^+^ and *Fgf17*^+^ RPC cells labeled approximately E9-E10 become broadly distributed in the telencephalon between E10.5 and E13.5 and ultimately generate neurons in the OB, rostral cortex, septal region, and basal ganglia. Notably, *Fgf8*^+^ progenitors from the RPC give rise to most cholinergic neurons in the basal forebrain. Compared to the Fgf8 lineage, the Fgf17 lineage generates fewer, more spatially restricted neurons. Fgf mutant studies revealed that Fgf8 and Fgf17 both impact the development of RPC progenitor populations. These findings provide novel insights into mechanisms that underlie Fgf functions, regional morphogenesis, and cellular complexity in the rostral telencephalon.

## Results

### Generation and validation of *Fgf8*^*CreER*^ and *Fgf17*^*CreER*^ knock in mice

**Figure 1:**
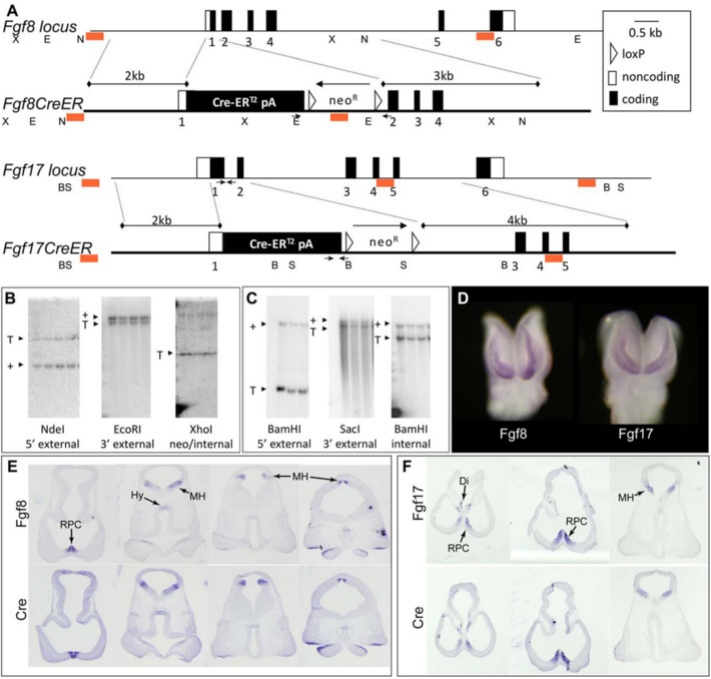
**Generation and validation of** ***Fgf8*** ^***CreER***^ **and** ***Fgf17*** ^***CreER***^ **mouse lines. (A)** In the *Fgf8* ^*CreER*^ allele, the first 20 nucleotides of exon 1 coding sequence were replaced with CreER-SV40 pA-loxP-neo-loxP cassette, and the long arm of homology began 12 nucleotides upstream of the exon 1/intron 1 junction. In the *Fgf17* ^*CreER*^ allele, all exon 1 coding sequences were replaced with CreER-SV40 pA. All Fgf17 intronic sequences were included in the targeted allele, but intron 2 was interrupted by insertion of the loxP-neo-loxP cassette and, further downstream, by a small insertion of MCS restriction enzyme sites. Symbols: small arrows, genotyping oligos; red rectangles, Southern blot probes. **(B)** *Fgf8* ^*CreER/+*^ and **(C)** *Fgf17* ^*CreER/+*^ Southern blots demonstrating correct targeting. **(D)** *Fgf8* and *Fgf17* whole mount ISHs (frontal view) showing mRNA expression in the rostral telencephalon of 12-s embryos. **(E,F)** E10.5 ISHs (horizontal sections) comparing *Fgf8 versus Cre* mRNA in an *Fgf8* ^*CreER/+*^ brain **(E)**, and *Fgf17 versus Cre* mRNA expression in an *Fgf17* ^*CreER/+*^ brain **(F)**. Sequential panels in **(E)** and **(F)** show successively more caudal planes of section. Abbreviations: B, BamHI; E, EcoRI; N, NdeI; S, SacI; X, XhoI; Di, diencephalon; MH, midbrain/hindbrain patterning center; Hy, hypothalamus. See also Additional file 1: Figure S1.

To characterize the lineages of *Fgf8*^*+*^*versus Fgf17*^+^ RPC cells, we generated mice harboring *Fgf8*^*CreER*^ and *Fgf17*^*CreER*^ knock in alleles, which are null for *Fgf8* and *Fgf17* and express inducible Cre under the control of endogenous regulatory elements (Figure 1A). We used CreER^T2^, a variant of the tamoxifen (Tm)-inducible CreER^Tm^ with higher Tm sensitivity [23], to maximize recombination efficiency and minimize Tm toxicity. Previous studies showed that Tm serum levels peak in adult mice 3 to 6 h post-administration and have an approximately 12-h half-life [24]. We and others found that *in utero*, Tm exposure activates CreER^Tm^ sparsely after 6 to 12 h but quite pervasively by 24 h; CreER^Tm^ is no longer active 48 h post-Tm (data not shown and [25]).

**Figure 2:**
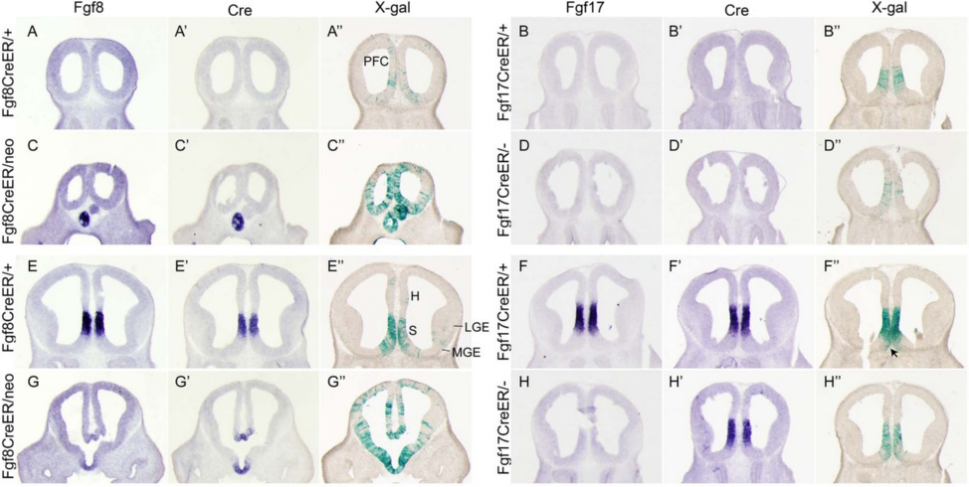
**Distribution of Fgf lineage cells at E12.5.** Comparison of *Fgf* (ISH), *Cre* (ISH), and βgal (Xgal stain) expression in E12.5 rostral **(A-D,A′-D′,A″-D″)** and caudal **(E-H,E′-H′,E″-H″)** coronal sections through the telencephalons of *ROSA26R* embryos (Tm E8.5). Note that there are Xgal^+^ cells rostral to the *Fgf* expression domains (we do not see *Fgf8* or *Fgf17* expression but we do see Xgal labeling in **A-D**″), and that in more caudal sections, Xgal^+^ cells are observed outside the *Fgf* expression domains **(E-H″)**. Abbreviations: ob, olfactory bulb neuroepithelium; h, hippocampus; mge, medial ganglionic eminence; pfc, prefrontal cortex; s, septum. See also Additional file 2: Figure S2.

Southern blots confirmed correct targeting, and *in situ* hybridizations (ISHs) confirmed that *CreER*^*T2*^ was expressed in *Fgf8* and *Fgf17* expression domains of *Fgf8*^*CreER*^ and *Fgf17*^*CreER*^ embryos, respectively (Figures 1, 2E-E′, and F-F′). Neomycin (neo) resistance cassettes were excised from knock-in alleles by crossing *Fgf8*^*CreER/+*^ and *Fgf17*^*CreER/+*^ (neo^+^) mice to *β-actin:: Cre* mice (data not shown; [26]). In pilot studies, tamoxifen (Tm) administration induced significantly more Cre reporter recombination in neo^−^ (compared to neo^+^) heterozygous knock-in embryos (data not shown). Hence, we used neo^−^ mice for all further experiments.

**Figure 3:**
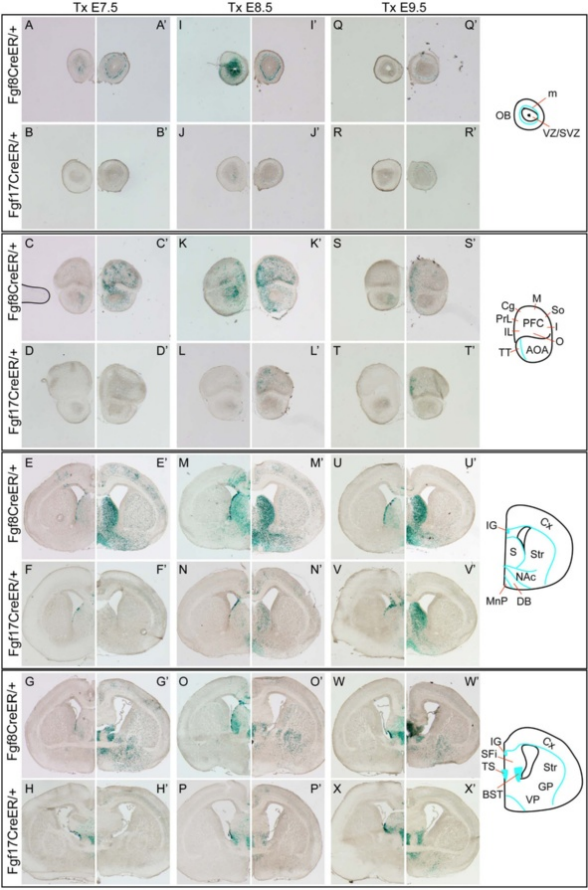
**Comparison of Fgf8 and Fgf17 fate maps.** Comparison of Fgf8 and Fgf17 fate maps in which progenitors were labeled at neural plate, early neurula, and late neurula stages. Xgal-stained coronal sections through E18.5 forebrains of *Fgf8* ^*CreER/+*^ and *Fgf17* ^*CreER/+*^ embryos on the **(A-X)** *ROSA26R* and **(A′-X′)** *TauR* backgrounds. Tm was administered at **(A-H, A′-H′)** E7.5, **(I-P, I′-P′)** E8.5, or **(Q-X, Q′-X′)** E9.5, marking cells from E8-E9.5, E9-E10.5, and E10-E11.5, respectively (see Experimental procedures). Abbreviations: m, mitral cell layer; VZ/SVZ, ventricular/subventricular zone; AOA, anterior olfactory area; TT, taenia tecta; Cx, cortex; IG, indusium griseum; S, septum; Str, striatum; NAc, nucleus accumbens; DB, diagonal band of Broca; MnP, median preoptic area; SFi, septofimbrial nucleus (septum); TS, triangular septal nucleus; BST, bed nucleus of the stria terminalis; GP, globus pallidus; VP, ventral pallidum. *PFC/Cortex areas:* Cg, cingulate; I, insular ; IL, infralimbic; M, motor; O, orbital; PrL, prelimbic; So, somatosensory.

**Figure 4:**
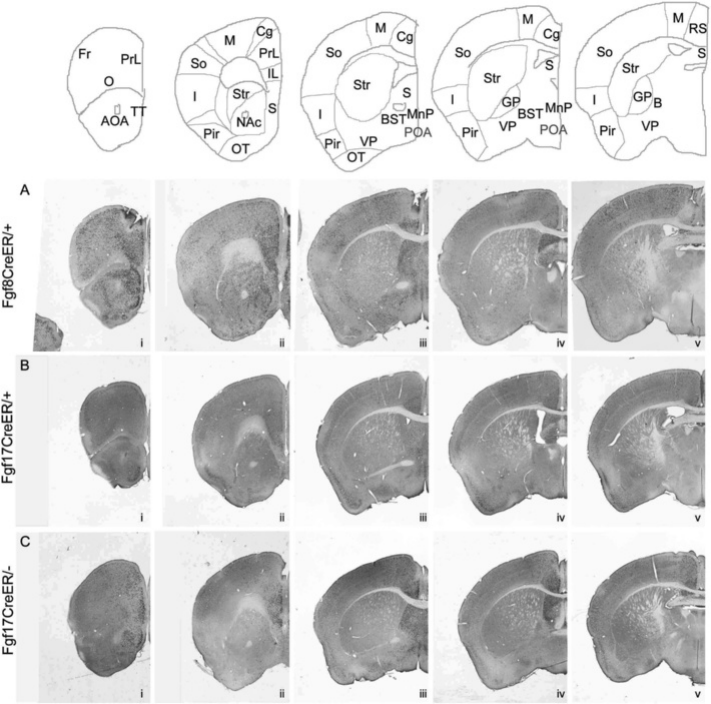
**Fates of Fgf8** ^**+**^ **and Fgf17** ^**+**^ **RPC cells in adult forebrains.** Anti-βgal IHC on coronal sections through **(A)** *Fgf8* ^*CreER/+*^; *TauR*, **(B)** *Fgf17* ^*CreER/+*^; *TauR*, and **(C)** *Fgf17* ^*CreER/-*^; *TauR* forebrains at P40 (Tm E8.5). Abbreviations (see also Figure 2 legend): B, nucleus basalis of Meynert; POA, preoptic area; Fr, frontal cortex; Pir, piriform cortex; RS, retrosplenial cortex; OT, olfactory tubercle. *Septum*: Both *Fgf8* ^*+*^ and *Fgf17* ^*+*^ progenitors give rise to cells in the lateral septum (LS), medial septum (MS), septofimbrial and septohypothalamic nuclei, triangular septal nucleus, and indusium griseum. The MS, which receives substantial contribution from the MGE [37], contained fewer labeled cells than other septal regions. *Cortex*: *Fgf8* ^*+*^ lineage cells were observed in the PFC and in most neocortical regions but were most concentrated in rostrodorsal areas (medial and dorsal frontal cortex, PrL, IL, Cg, M, RS). In contrast, *Fgf17* ^*+*^ lineage cells only populated the medial PFC and the Cg, M, and dorsal S areas of the neocortex. *Basal ganglia*: The *Fgf8* ^*+*^ lineage, but not the *Fgf17* ^*+*^ lineage, makes prominent contributions to the striatum, ventral pallidum, accumbens, and globus pallidus. We observed left/right asymmetries in *Fgf8* ^*+*^ and *Fgf17* ^*+*^ fate maps that were particularly striking in the neocortex, shown in Additional file 3: Figure S3. See also Figure 3.

**Figure 5:**
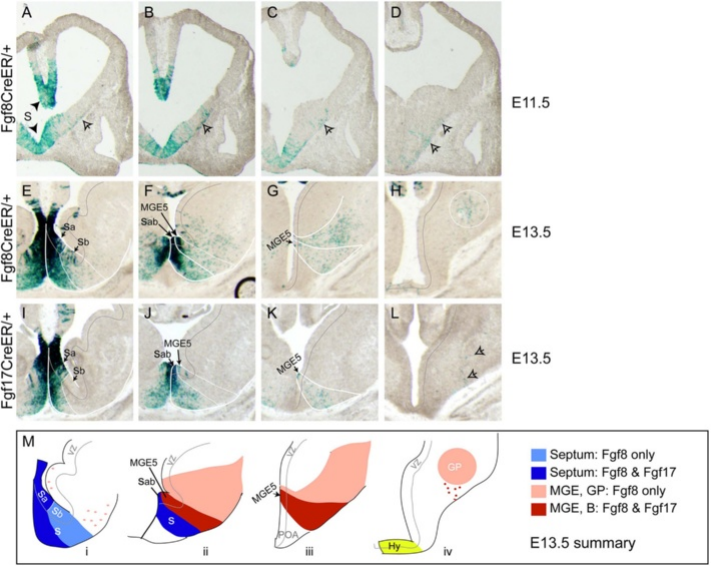
**Origins of Fgf lineage cells in the basal ganglia.** Xgal stained coronal sections through caudal telencephalons of **(A-D)** E11.5 *Fgf8* ^*CreER/+*^; *ROSA26R*, **(E-H)** E13.5 *Fgf8* ^*CreER/+*^; *ROSA26R*, and **(I-L)** E13.5 *Fgf17* ^*CreER/+*^; *ROSA26R* embryos (Tm E8.5). **(M)** Diagram illustrating similarities and differences between E13.5 fate maps. In panels **(F)**, **(J)**, and (Mii), S_ab_ indicates that we are unable to distinguish S_a_ and S_b_; the dark blue septal field extending from S_ab_ in (Mii) likely contains cells from both progenitor domains. As shown in **(H)** and **(L)**, the hypothalamus contains *Fgf8* ^*+*^ but not *Fgf17* ^*+*^ lineage cells at E13.5. Arrows in **(A-D)** indicate cells concentrated near the pial surface that appear to be migrating dorsally and caudally from the septum and vMGE. Arrowhead in **(L)** indicates cells from this pial migratory stream that appear to migrate radially toward the nucleus basalis of Meynert. Lateral striatum cells in the *Fgf8* ^*+*^ lineage, indicated in pink in (Mi), are likely be derived from S_b_. Abbreviations: S, septum; POA, preoptic area; Hy, hypothalamus; B, nucleus basalis of Meynert. See also Additional file 5: Figure S5.

We used both *ROSA26R* [27] and *Tau*^*lox-STOP-lox-mGFP-IRES-NLS-LacZ-pA*^ (*TauR*; [28]) Cre reporters. We found that *ROSA26R* (used for embryonic studies) marks neural progenitors but is not active in all mature neurons, whereas *TauR* (used in peri- and postnatal studies) efficiently labels neurons but not progenitors (Figures 2, 3, 4, and 5 and data not shown).

### RPC progenitor subtypes labeled in early neurulae differentially contribute to multiple telencephalic structures

Fgfs are dynamically expressed in the early RPC. Fgf8, detectable at E8.5, precedes Fgf17 expression in the RPC [29,17]. From E9.0 (12 somites) to E9.5, Fgf8 and Fgf17 are expressed in similar RPC domains (Figure 1D, Additional file 1: Figure S1A-E; [29,17]). However, by E10.5, *Fgf17* expression extends farther from - and may be excluded from - the *Fgf8*^*+*^ midline (Additional file 1: Figure S1G,I; [13]). To compare the lineages of RPC cells labeled at different stages, we evaluated *Fgf8*^*CreER/+*^ and *Fgf17*^*CreER/+*^ (*ROSA26R, TauR*) fate maps after Tm administration at E7.5, E8.5, and E9.5. Resultant fate maps indicated that the Fgf17 lineage labeled at successive stages gives rise to increasingly more cells of similar fate potential. At E18.5, Fgf17 lineage cells were predominantly restricted to the septum, diagonal band of Broca (DBB), olfactory bulb (OB) mitral cell layer, and dorsomedial cortex (Figure 3). The Fgf8 lineage labeled by Tm E7.5 or Tm E8.5 generated many more cells in these structures and also contributed to other neocortical areas, the basal ganglia, and the OB ventricular zone (VZ; Figure 3). These findings were surprising in light of a previous study that used *Fgf8-IRES-Cre; ROSA26R* mice and reported Fgf8 lineage cells only in the septum and medial PFC [14]. The competence of *Fgf8*^*+*^ progenitors to contribute to the cortex and OB declined in late neurulae, and so Fgf8 and Fgf17 Tm E9.5 fate maps were quite similar (though the Fgf8 lineage still generated more basal ganglia cells; Figure 3Q-X′).

These results demonstrate that Fgf^+^ RPC cells contribute to several telencephalic structures and suggest that *Fgf8* and *Fgf17* mark progenitor subpopulations in the E9-E10 RPC that originate in similar spatial domains but differ in their developmental potential. For all further experiments, we administered Tm at E8.5 to visualize Fgf8/17^+^ RPC lineages labeled at the peak of their potential.

### Distribution of RPC-derived progenitor cells during early telencephalon development

E18.5 fate maps indicated that the RPC is a source of progenitors for multiple telencephalon structures. To elucidate how progenitors reach these structures, we monitored their behavior during early telencephalon development.

**Figure 6:**
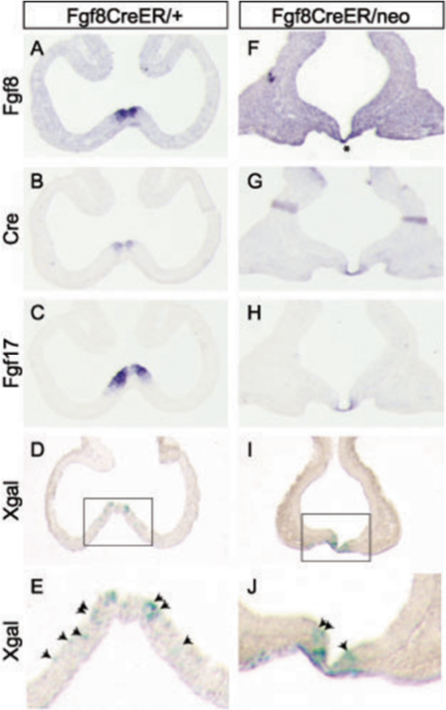
**Analysis of gene expression and fate maps in** ***Fgf8*** ^***CreER/neo***^ **embryos. (A,F)** *Fgf8*, **(B, G)** *Cre*, **(C, H)** *Fgf17*, and **(D, E, I, J)** Xgal staining in horizontal sections through E10.5 *Fgf8* ^*CreER/neo*^; *ROSA26R* and control embryos (Tm E8.5). **(J,K)** are high magnification images of boxed areas in **(D,E)**; arrowheads indicate Xgal^+^ cells outside the *Fgf8* ^*+*^ domain. ISH shown in **(F)** was left to develop for a long time in order to visualize the low level *Fgf8* expression in *Fgf8* ^*CreER/neo*^ embryos, hence the background on this section is quite high. Asterisk **(F)** marks the RPC, which is clearly visible in the Cre and Fgf17 ISHs. Note that Cre is expressed in the RPC but not elsewhere in the telencephalon (Horizontal lines in the caudal telencephalon in **(G)** are artifacts due to tissue folds).

In *Fgf8*^*CreER/+*^; *ROSA26R* embryos, Xgal^+^ cells were concentrated in the *Fgf8*^*+*^ RPC domain at E10.5, but were also observed sparsely in nearby *Fgf8*^*−*^ neuroepithelium (Additional file 1: Figure S1A-C,G,H). At E12.5, Fgf8 lineage cells were still most concentrated in the *Fgf8*^*+*^ septum but also mosaically populated the neuroepithelium of *Fgf8*^*−*^ structures including the presumptive OB, PFC, hippocampus, ventral medial ganglionic eminence (vMGE), and neocortex (Figure 2A-A″, E-E″, and data not shown). *In situ* hybridizations at multiple stages confirmed that *CreER*^*T2*^ mRNA was restricted to the *Fgf8*^*+*^ domain (ISH; Figures 1E, 2A-A′, E-E′, and 6A-B, and data not shown). These data support a model in which a subset of Fgf8 lineage progenitors disperse or are displaced within the VZ away from the RPC.

The *Fgf17*^+^ fate maps suggest that this RPC lineage has a more restricted developmental potential than the *Fgf8*^+^ lineage. At E10.5, we observed few if any *Fgf17*^+^ lineage cells lateral to the *Fgf17*^*+*^ domain (Additional file 1: Figure S1D-F, I, J). At E12.5, *Fgf17*^*+*^ lineage cells were confined in rostral sections to the ventromedial PFC; labeled cells were observed rostral to the RPC, but unlike the *Fgf8*^+^ lineage cells (Figure 2A″), the *Fgf17*^*+*^ lineage was quite restricted in its dorsal/ventral distribution (Figure 2B″,F″). In caudal sections, *Fgf17*^*+*^ lineage cells remained in the *Fgf17* mRNA-positive domain except for a small population of cells extending ventromedially to the pial surface (arrow in Figure 2F″). At E13.5, the difference in *Fgf8*^*+*^*versus Fgf17*^*+*^ lineage dispersion was even more pronounced: there were more *Fgf8*^*+*^ (*versus Fgf17*^*+*^) lineage cells in the OB, rostral cortex, ventral septum, and vMGE (Additional file 2: Figure S2A-B and not shown).

Thus, *Fgf8*^*+*^ and *Fgf17*^*+*^ lineage progenitors, which originate from a similar domain in the RPC, exhibit distinct developmental potential during early forebrain development and consequently are poised to contribute cells to overlapping and distinct telencephalon structures.

### Neuronal derivatives of *Fgf8*^*+*^ and *Fgf17*^*+*^ RPC progenitors

Next, we conducted E18.5 and P40 fate map and co-labeling studies to characterize neuronal derivatives of *Fgf8*^*+*^ and *Fgf17*^*+*^ RPC cells in the mature telencephalon and to gain more insight into the distinctions between these lineages.

The septum, which forms around the vestiges of the RPC in the rostromedial telencephalon, contained the highest density of labeled cells in P40 *Fgf8* and *Fgf17* fate maps (Figure 4; Additional file 3: Figure S3E, F). The *Fgf8*^*+*^ lineage contributed the majority of each septal neuronal subtype examined in co-localization studies (Table 1, Additional file 4: Figure S4J-O). For most subtypes, the *Fgf17*^*+*^ lineage co-labeled with approximately 30% as many cells as the *Fgf8*^*+*^ lineage. The exception to this was the Ctip2^+^ subpopulation, of which approximately 70% were labeled by *Fgf8*^*CreER*^ and only approximately 10% by *Fgf17*^*CreER*^. The diagonal band of Broca (DBB) also received prominent contributions from *Fgf8*^*+*^ and *Fgf17*^*+*^ lineages, likely due to short-range ventral migration of RPC-derived cells. *Fgf8*^*+*^ lineage DBB cells were superficial to the CTIP2^+^ domain and included most Tbr1^+^ and cholinergic (ChAT^+^) cells, approximately 50% of calbindin^+^ cells, and approximately 30% of Nkx2.1^+^ cells (Table 1, Additional file 4: Figure S4P-S, data not shown). *Fgf17*^*CreER*^ labeled roughly the same proportion of Nkx2.1^+^ cells but lower proportions of other subtypes in the DBB (Table 1, data not shown).

**Table 1:** **Confocal analysis of Fgf lineage cells at E18.5 and P40**

			***Fgf8*** ^***CreER/+***^			***Fgf17*** ^***CreER/+***^		
**Structure**	**Age**	**Marker**	**Double positive/βgal** ^**+**^	**Double positive/marker** ^**+**^	***n***	**Double positive/βgal** ^**+**^	**Double positive/marker** ^**+**^	***n***
*Cortex*	P40	PV (medial)	2%	10%	2	0%	0%	2
	P40	PV (lateral)	4%	12%	2	3%	1%	2
	P40	SS (medial)	2%	6%	2	0%	0%	2
	P40	SS (lateral)	6%	17%	2	0%	0%	2
*Septum*	E18.5	Calbindin	2%	60%	1	17%	23%	1
	E18.5	Calretinin	16%	58%	1	24%	19%	1
	E18.5	Ctip2	24%	69%	1	15%	9%	1
	E18.5	Nkx2.1	5%	74%	1	19%	20%	1
	P40	ChAT (MS)	8%	77%	2	17%	33%	4
*Diagonal band of Broca*	E18.5	Calbindin	10%	50%	1	6%	19%	1
	E18.5	Nkx2.1	2%	33%	1	9%	26%	1
	E18.5	Tbr1	34%	95%	1	59%	41%	1
	P40	ChAT	13%	80%	2	28%	44%	4
*Striatum*	P40	ChAT	23%	66%	3	64%	16%	3
	P40	Ctip2	70%	9%	3	nd	nd	nd
	P40	PV	1%	14%	2	0%	0%	2
	P40	SS	2%	29%	2	nd	nd	nd
*Globus pallidus*	E18.5	Ctip2	96%	35%	1	71%	3%	1
	E18.5	Nkx2.1	92%	37%	1	89%	4%	1
	P40	Npas1	72%	28%	3	88%	5%	2
	P40	PV	46%	38%	2	0%	0%	2
*Nucleus basalis of Meynert*	P40	ChAT	49%	75%	3	92%	43%	3
*Ventral pallidum*	P40	ChAT	42%	76%	3	95%	45%	3

Quantification of confocal studies examining co-localization of βgal with cell type-specific markers in *Fgf8* ^*CreER/+*^; *TauR* and *Fgf17* ^*CreER/+*^; *TauR* animals (Tm E8.5). Parenthetical descriptors (lateral, medial, MS) indicate regional location within the structure. See also Additional file 4: Figures S4 and Additional file 5: Figure S5. Cortical interneuron counts are overestimates, as quantification was done only in regions containing βgal^+^ cells.

Outside the septum, *Fgf*^*+*^ RPC lineage cells populated E18.5 *ROSA26R* VZs with varying, structure-dependent degrees of mosaicism that reflected early biases in progenitor distribution. As predicted by E12.5 and E13.5 fate maps, in which many *Fgf8*^*+*^ lineage (and few *Fgf17*^*+*^ lineage) cells were observed at the rostral pole, the *Fgf8*^*+*^ lineage generated many neurons in the OB, accessory olfactory bulb, and anterior olfactory area (AOA). *Fgf17*^*+*^ lineage cells were scarce in these structures (Figure 3A-B, Figure 4A-B, Additional file 4: Figure S4A-D). In the OB, *Fgf8*^*+*^ lineage cells included glutamatergic mitral cells as well as GABAergic periglomerular and granule cells (Additional file 4: Figure S4A-D). We also observed sparse Fgf8 lineage interneurons (somatostatin (SS)^+^) in the external plexiform layer and the intrabulbar part of the anterior commissure (data not shown). In the AOA, *Fgf8*^*+*^ lineage cells were distributed in a medial > lateral gradient (the few *Fgf17*^*+*^ lineage cells were restricted medially; Figure 4Ai, Bi). Fewer than 5% of *Fgf8*^*+*^ lineage AOA cells were interneurons, comprising up to 30% of PV^+^ and 6% of SS^+^ cells (data not shown).

*Fgf8*^*+*^ lineage cells contributed more prominently to the neocortex than *Fgf17*^*+*^ lineage cells. Both lineages were most concentrated in rostrodorsal cortical areas, but *Fgf8*^*+*^ lineage neurons were also observed in ventral areas (Figures 3 and 4A, B). *ROSA26R* fate maps suggested that most neocortical cells were projection neurons: there were very few *Fgf8*^*+*^*/Fgf17*^*+*^ lineage cells in the MGE VZ (origin of cortical interneurons) but numerous *Fgf8*^*+*^ (fewer *Fgf17*^*+*^) lineage clones extending radially from the VZ into the cortical plate, as expected for projection neurons (Figure 3M-N, Additional file 2: Figures S2A-B, Additional file 3: S3A-B′). Indeed, many *Fgf8*^*+*^ and *Fgf17*^*+*^ lineage cortical neurons expressed projection neuron markers (Tbr1, Ctip2), and very few expressed interneuron markers (PV, SS; Table 1, Additional file 4: Figure S4E-I, data not shown).

The *Fgf8*^*+*^ lineage also contributed many more cells than the *Fgf17*^*+*^ lineage to the basal ganglia. The sparse *Fgf17*^*+*^ lineage cells in the striatum, nucleus basalis of Meynert, and ventral pallidum were strongly biased to cholinergic (ChAT^+^) fates (Table 1, data not shown). The *Fgf8*^*+*^ lineage gave rise to most basal ganglia cholinergic cells in these areas but also generated several other cell types (Figures 3 and 4, Table 1, Additional file 5: Figure S5I). In the striatum, the *Fgf8*^*+*^ lineage generated subpopulations of medium spiny neurons (Ctip2^+^; [30]), SS^+^, and PV^+^ interneurons (Table 1, Additional file 5: Figure S5A-D, data not shown). In the GP, the few *Fgf17*^*+*^ lineage neurons were mostly NPAS1^+^, whereas the *Fgf8*^*+*^ lineage included Nkx2.1^+^, Ctip2^+^, PV^+^, and NPAS1^+^ neurons (Table 1 and Additional file 5: Figure S5E-H).

Overall, these data demonstrate that these RPC lineages generate many neuronal subtypes throughout the rostral telencephalon, but the *Fgf17*^*+*^ lineage is more spatially restricted and in some areas more fate-restricted than the *Fgf8*^*+*^ lineage.

### RPC-derived progenitor domains for the basal ganglia

*Fgf8*^*+*^ (and to a lesser extent, *Fgf17*^*+*^) lineage cells gave rise to prominent subpopulations of basal ganglia neurons but were only sparsely observed in expected basal ganglia progenitor domains (MGE/LGE VZs; Figures 3 and 5M). We scrutinized embryonic fate maps to identify RPC-derived progenitor domains and migratory routes that would explain the development of *Fgf8*^*+*^ and *Fgf17*^*+*^ lineage basal ganglia neurons.

At E11.5, *Fgf8*^*+*^ lineage VZ cells were most concentrated in the medial/ventral septum and were observed at lower density in the MGE (Figure 5A-D). Labeled cells from these progenitor domains appeared to migrate dorsally and caudally in the marginal zone toward the LGE and caudal MGE (arrows, Figure 5A-C). Indeed, The caudal MGE had very few *Fgf8*^*+*^ lineage VZ cells but did contain *Fgf8*^*+*^ lineage cells in their mantle zones (Figure 5D, arrows).

E13.5 fate maps suggested that at least three distinct progenitor domains in the septum and vMGE give rise to most subpallial *Fgf*^*+*^ lineage cells. We identified two etiologically and functionally distinct progenitor domains in the ventral septum VZ (previously designated Se4; [31]) based on contributions from the *Fgf8*^*+*^ and *Fgf17*^*+*^ lineages. The dorsomedial domain, S_a_, was densely populated by both *Fgf8*^*+*^ and *Fgf17*^*+*^ lineages and gave rise to cells that were restricted to the ventromedial mantle zone and pial surface (Figure 5E,I,M). These cells likely contribute to the DBB and ventral pallidum. In contrast, the more ventral domain, S_b_, included *Fgf8*^*+*^ but not *Fgf17*^*+*^ lineage cells (Figure 5E,I,M). S_b_-derived cells were more diffuse and migrated ventrally as well as dorsally, likely generating striatal cells (Figure 5E,F). More caudally, we observed many *Fgf8*^*+*^ and *Fgf17*^*+*^ lineage cells in the MGE5 progenitor domain [31]; MGE5-derived cells appeared to migrate ventrolaterally and caudally toward the ventral pallidum and nucleus basalis of Meynert (Figure 5F-H, J-M). The *Fgf8*^*+*^ (but not *Fgf17*^*+*^) lineage also generated a large population of cells that appeared to migrate laterally out of dorsal MGE5 or ventral MGE4 to generate neurons in the striatum, GP, and bed nucleus of the stria terminalis (Figure 5E,F, pink in 5Mii, iii). These findings, together with our co-localization data, provide novel insights into the origins of select basal ganglia subpopulations.

### Fgf signaling impacts the development of RPC-derived progenitors

Many telencephalon structures affected in *Fgf8* and *Fgf17* mutant mice received prominent contributions from the *Fgf8*^*+*^ and *Fgf17*^*+*^ RPC lineages, respectively [12,7,10,8,2,6,9,3,5,4,11]. Thus, we hypothesized that mutant phenotypes may in part reflect Fgf roles in the development of RPC-derived progenitors. To investigate this, we conducted lineage studies in *Fgf8*^*CreER/neo*^ (that is, *Fgf8* hypomorphs) and *Fgf17*^*CreER/-*^ (that is, *Fgf17* null) embryos. We used *Fgf8*^*CreER/neo*^ embryos, which express very low levels of Fgf8, because *Fgf8*^*−/−*^ embryos die prior to telencephalon development [32,33]. *Fgf8*^*CreER/neo*^ embryos have variable phenotypes; we selectively used embryos that did not have severe, early morphogenetic phenotypes.

At E10.5, *Fgf8*^*+*^ lineage cells behaved similar in *Fgf8*^*CreER/neo*^ and control embryos: Xgal^+^ cells were predominantly restricted to the *Fgf8*^*+*^ RPC domain, with a small amount of lateral dispersion (Figure 6E,J). However, at E12.5 and E13.5, *Fgf8*^*CreER/neo*^ embryos had many more Xgal^+^ cells than controls, and labeled cells were distributed ectopically throughout pallium and subpallium (Figure 2, Additional file 3: Figure S2E). This was not due to ectopic expression of CreER: *Fgf8* and *CreER* transcripts remained restricted to the appropriate RPC subdomain in *Fgf8* hypomorphs (Figures 2 and 6). *Fgf17*^*+*^ lineage cells also behaved abnormally on the *Fgf8*^*neo/−*^ background. Though not as widely dispersed as *Fgf8*^*+*^ lineage cells, there were more *Fgf17*^*+*^ lineage cells in the rostral and dorsomedial cortex and the vMGE of *Fgf17*^*CreER/+*^; *Fgf8*^*neo/−*^ embryos at E13.5 (Additional file 2: Figure S2F). There was also a marked reduction of *Fgf17*^*+*^ lineage cells in the presumptive septum (Additional file 2: Figure S2F). These data support the conclusion that Fgf8 negatively regulates the dispersion of RPC-derived progenitors between E10.5 and E13.5. The differences between *Fgf17*^*CreER/+*^, *Fgf8*^*neo/-*^, and *Fgf8*^*CreER/neo*^ fate maps further support the hypothesis that Fgf8 and Fgf17 mark functionally distinct RPC progenitor subtypes.

In parallel experiments, we found that *Fgf17*^*CreER/−*^ embryos appeared to have slightly fewer Xgal^+^ cells than control littermates at E12.5 and E13.5 (Figure 2, Additional file 2: Figure S2). In these *Fgf17*^*CreER/−*^ mutants, *Fgf17*^*+*^ lineage cells were also more broadly distributed in the PFC, dorsomedial, and ventrolateral cortices (Figure 2D″, Additional file 2: Figure S2Diii). The *Fgf8*^*+*^ lineage was also aberrant on the *Fgf17*^*−/−*^ background: it gave rise to fewer ventral/lateral septal cells and more cells in the rostral MGE and caudal dorsomedial cortex, relative to controls (Additional file 2: Figure S2C). Although these E12.5-E13.5 phenotypes were very subtle, adult (P40) fate maps revealed that the *Fgf17*^*+*^ lineage was expanded in the OB, AOA, septum, PFC, rostral neocortex, and striatum of *Fgf17*^*CreER/−*^ mice (Figure 4B,C).

Thus, analysis of the mutant fate maps demonstrates that Fgf8 and Fgf17 impact the number and localization of RPC-derived progenitors during early telencephalon development, and that Fgf8 has a stronger effect on this process than Fgf17.

### Attempts to define the mechanism(s) underlying the Fgf8 hypomorphic fate map phenotype

The Fgf8 hypomorphic mutant fate map phenotype becomes apparent between E10.5 and E11.5, when labeled Fgf8^+^ lineage cells also begin to populate non-RPC structures in control embryos. We conducted a series of experiments aimed to elucidate the molecular mechanism underlying the mutant phenotype.

First, we performed control fate map experiments to determine whether CreER activity outside the RPC may contribute to the broad distribution of labeled cells in *Fgf8*^*CreER/+*^ fate maps and/or the mutant fate map phenotype. This could be due to ‘leaky’ CreER activity or to sporadic, transient Fgf8 expression in the VZ that has not previously been recognized due to timing and/or levels. However, in ‘no tamoxifen’ control experiments, we did not observe Tm-independent CreER activity that could explain the distribution of Fgf8^+^ lineage cells outside the RPC or the Fgf8 hypomorphic fate map phenotype (data not shown).

Next, to examine CreER activity between E10.5 and E11.5, we administered Tm at E10.5 and examined E12.5 ROSA26R fate maps. In these experiments, we did not see any evidence for CreER activity outside the RPC (data not shown). These data, together with the fate maps that resulted from E8.5 and E.9.5 Tm administration (discussed earlier), do not support a model in which the mosaic distribution of Fgf8^+^ lineage cells and the aberrant distribution in mutant embryos results from ectopic CreER activity.

Then, we investigated the hypothesis that the Fgf8 hypomorphic fate map phenotype was due to an increase in RPC progenitor proliferation in Fgf8 hypomorphs, which in turn could lead to an increase in the number of labeled cells and/or enhanced displacement of cells away from the RPC. However, our analysis of proliferation at E10.5 using antibody staining of the M-phase marker, phosphohistone-3 (data not shown), supported published findings (Storm *et al*., 2006) that the Fgf8 hypomorphs have reduced proliferation in the rostral telencephalon VZ.

**Figure 7:**
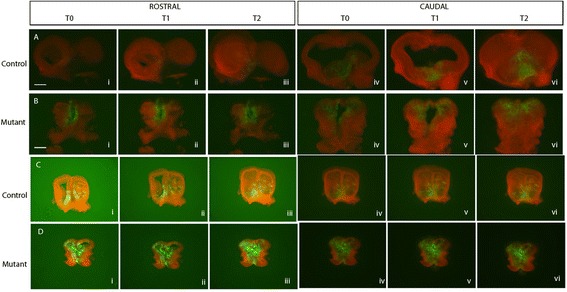
**Time lapse imaging of Fgf8** ^**+**^ **lineage cells in brain slice culture.** *Fgf8* ^*CreER/+*^ and *Fgf8* ^*CreER/neo*^ mice on an mT/mG background exposed to tamoxifen at E8.5. At either E10.5 (panels in rows (**A**,**B**)) or E11.5 (panels in rows (**C**,**D**)), their brains were removed, sectioned on a vibratome and grown in slice culture [(neurobasal media (NBM)]. Slices were photographed approximately every 12 h, and representative images are shown. Cells in which the reporter *had not* undergone Cre-mediated recombination are red, recombined cells are green. The brains shown are from litters administered with tamoxifen and dissected in parallel. ‘Rostral’ sections, through the RPC, are in the left panels; ‘caudal’ sections, through the level of containing the MGE, are in the right panels. T0 (i, iv) = onset of culture; T1 (ii, v) = approximately 24 h in culture; T2 (iii, vi) = approximately 48 h in culture. Note that the mutant had widely distributed green cells even at T = 0, and that their distribution did not appear to change during culture.

Finally, toward assessing whether reduced Fgf signaling increases lateral dispersion/migration of cells from the RPC, we assessed cell movements by time lapse imaging of control (*Fgf8*^*CreER/+*^) and mutant (*Fgf8*^*CreER/neo*^) embryonic brain slice cultures. For these experiments, we used the dual reporter mT/mG reporter mouse, in which cells express tandem dimer tomato (a red fluorophore) in the absence of Cre and green fluorescent protein (GFP) after Cre-mediated recombination (Muzumdar *et al*., 2007). Tm was administered to the mice at E8.5, their brains were isolated at E10.5 or E11.5, then sectioned coronally and grown as slice cultures. The distribution of green cells was assessed used time lapse imaging. As shown in Figure 7, the distribution of labeled Fgf8 lineage (green) cells in this system was very similar to that observed using the ROSA26R and TauR reporters (Figure 2C″,G″ and Additional file 2: Figure S2E). Importantly, the distribution of green cells did not change between T0 and T2 (approximately 48 h), providing evidence that there was no tangential spread of Fgf-lineage cells after E10.5, in control or mutant brains.

## Discussion

### The RPC is an important source of telencephalic progenitors

Our lineage analyses demonstrated that progenitors originating in the RPC mosaically populate different telencephalon structures during early forebrain development. This may be a mechanism by which distinct progenitor subtypes from the RPC and other sources become intermingled and/or poised to generate various neuronal subtypes in different structures. Indeed, we have shown that RPC-derived cells give rise to multiple neuronal subtypes in the OB, neocortex, basal ganglia, and septum. Furthermore, if Fgf8/17 protein secretion perdures after progenitors leave the RPC, then dispersion of RPC-derived cells could impact Fgf protein localization in the early telencephalon. This suggests a novel, cell-based mechanism that could contribute to formation of the forebrain’s Fgf8 protein gradient (previously attributed to Fgf diffusion; [14]).

The mechanism by which RPC-derived cells become distributed throughout the forebrain remains unclear. Time-lapse studies tracking cell division and movement in embryonic brain slices did not support a mechanism of active migration away from the RPC. Instead, it is possible that cell division and intercalation passively displace Fgf lineage cells away from the RPC as the telencephalon grows. This could account for the peak concentration of Fgf lineage cells in the septal anlage. However, we observed clear biases in the fate maps that are not explained by this passive mechanism: Fgf lineage cells appear to be excluded from select telencephalon regions and do not populate the telencephalon according to a simple gradient as predicted by a displacement model. Furthermore, this mechanism does not, in isolation, explain how molecularly distinct progenitor subtypes originating from approximately the same RPC domain differ significantly in their developmental potential. There may be guidance cues that attract, repel, or form boundaries that shape the distribution of RPC-derived progenitors in the early telencephalon. *Fgf8*^*+*^ and *Fgf17*^*+*^ progenitors may respond differently to such cues, and the difference in the number of *Fgf8*^*+*^*versus Fgf17*^*+*^ progenitors may be due to distinct proliferative behaviors.

Our results differ in part from a previous study that reported Fgf8 lineage cells to be restricted to the septum and medial frontal cortex [14]. The discrepancies between our results and those of Toyoda *et al*. are likely due to differences in experimental methodology. First, the previous study used an allele in which Cre coding sequences followed an IRES. Consequently, Cre may have not been expressed at sufficient levels in all *Fgf8*^*+*^ cells to report the complete fate map. In our allele, CreER is expressed directly from the Fgf8 promoter, and we demonstrated that CreER and Fgf8 are co-expressed in all domains examined in heterozygous embryos. Second, our fate maps were performed using mice that were heterozygotes for loss of *Fgf8* or *Fgf17* function. While we have not observed a molecular or anatomic phenotype caused by the reduced gene dosage, in principle it is a possibility. The *Fgf8-Cre* allele used by Toyoda *et al*. should have normal Fgf8 gene dosage. Third, Toyoda *et al*. reported only E10.5 and E14.5 *ROSA26R* fate maps, whereas we used *ROSA26R* and *TauR* and analyzed E10.5-E13.5, E18.5, and P40 fate maps. Our perinatal and postnatal *TauR* fate maps were instrumental in identifying neuronal derivatives of the Fgf8 lineage, especially in the basal ganglia, OB, and cortex.

### Fgf8^+^ and Fgf17^+^ progenitors differ in their developmental competence and are transiently able to contribute to several telencephalic structures

Our experimental approach allowed us to visualize comprehensive progenitor and neuronal fate maps of *Fgf8*^*+*^ and *Fgf17*^*+*^ RPC progenitors. Importantly, we demonstrated that *Fgf8* and *Fgf17* mark closely apposed yet functionally distinct progenitor populations in the RPC. Using Tm-regulated knock-in Cre lines, we evaluated how the potential of these progenitor subtypes change during early telencephalon development. We found that the developmental competence of *Fgf8*^*+*^ and *Fgf17*^*+*^ cells does not change significantly from the onset of RPC Fgf expression (Fgf8: E8.0-E8.5, Fgf17: E8.75-E9.0) through the early/mid-neurula stage (approximately E9.5). *Fgf8*^*+*^ and *Fgf17*^*+*^ lineages labeled during this time both make prominent contributions to the dorsomedial PFC, septum, DBB, OB mitral cell layer, and telencephalic cholinergic populations. However, the *Fgf8*^*+*^ lineage is more broadly distributed and constitutes more cells than the *Fgf17*^*+*^ lineage (except perhaps in the cingulate cortex). Furthermore, *Fgf8*^*+*^ but not *Fgf17*^*+*^ progenitors labeled during this period make robust contributions to the OB granule cell and periglomerular cell layers, ventral neocortex, striatum, and globus pallidus. By late neurula stages (E10-E11.5), *Fgf8*^*+*^ progenitors lose their competence to generate neocortical and OB progenitors. The *Fgf8*^*+*^ lineage labeled at this time still made more prominent contributions to the basal ganglia, but otherwise the *Fgf8*^*+*^ and *Fgf17*^*+*^ lineages behaved similarly and were both predominantly restricted to the septum, DBB, medial PFC, and ventral pallidum. These results were surprising given the broader domain of *Fgf17* expression at E10.5.

Differences between *Fgf8* and *Fgf17* fate maps could be explained by a model in which *Fgf8*^*+*^ RPC cells are especially pluripotent and/or motile prior to the onset of *Fgf17* expression, and the two ligands subsequently mark the same progenitors. Further experiments are needed to characterize the relationships between *Fgf8*^+^ and *Fgf17*^+^ cells in the RPC, such as a dual labeling approach that can simultaneously distinguish *Fgf8-* and *Fgf17*-expressing cells. However, based on our fate mapping results, we hypothesize that a subset of *Fgf17*^+^ progenitors initially express *Fgf8*. Differences in the efficiency of tamoxifen-induced recombination between the *Fgf8*^*CreER*^ and *Fgf17*^*CreER*^ lines could contribute quantitative differences in the fate maps. Nonetheless, the comparative fate maps provide evidence that the two ligands partially mark distinct populations.

For instance, *Fgf8* and *Fgf17* are both strongly expressed in the RPC E9-E10.5, yet *Fgf8*^*+*^ cells labeled during this time (Tm E8.5) contribute more broadly than *Fgf17*^*+*^ lineage progenitors to the telencephalon. The *Fgf8*^*+*^ lineage also made more prominent contributions to the basal ganglia when labeled after E9.5, when *Fgf8* and *Fgf17* are both strongly expressed. Furthermore, co-labeling data indicated that *Fgf8*^*+*^ progenitors generate more neuronal subtypes in some regions, suggesting that *Fgf17*^*+*^ lineage cells may be more fate restricted in target tissues. Thus, we propose that *Fgf8* and *Fgf17* mark two subtypes of RPC progenitors that differ in their developmental potential, with *Fgf17*^*+*^ lineage cells from the RPC more restricted in their distribution and ultimate fates than *Fgf8*^*+*^ lineage cells. Of course, there are likely some progenitors that co-express both Fgfs. mRNA expression data (Figure 1 and [13]) suggest that unless RPC Fgfs are subject to post-transcriptional regulation, *Fgf8*^*+*^ and *Fgf17*^*+*^ RPC progenitors are intermixed in the early neurula and subsequently segregate to spatially distinct subdomains. Intriguingly, the medial RPC has a slower proliferative rate than neighboring cells at E9.0 as has been observed in other stem cell niches [10,34].

### Origins of Fgf^+^ lineage neurons in the basal ganglia

We observed sparse *Fgf8*^*+*^ lineage cells in the LGE VZ that likely give rise to approximately 10% of striatal MSNs (Table 1, [35]). However, most *Fgf8*^*+*^*/17*^*+*^ lineage cells in the basal ganglia appeared to originate in the septum and vMGE progenitor domains. Based on our results, we propose the following model (Figure 5M). As the telencephalon grows, the RPC expands in the medial septum VZ and includes S_a_, a source of *Fgf8*^*+*^ and *Fgf17*^*+*^ lineage progenitors that give rise to DBB and ventral pallidum neurons. *Fgf8*^*+*^ lineage cells disperse ventrolaterally to S_b_, which generates striatal neurons; *Fgf17*^*+*^ lineage cells are excluded from S_b_ by a ventral dispersion boundary. *Fgf8*^*+*^ and *Fgf17*^*+*^ lineage cells disperse caudally and laterally from S_a_ and S_b_, respectively, to MGE5. MGE5-derived cells migrate ventrolaterally and caudally to generate ventral pallidum and nucleus basalis of Meynert neurons. *Fgf8*^*+*^ but not *Fgf17*^*+*^ lineage cells may also populate a subdomain of MGE5/MGE4 that gives rise to striatum and GP neurons.

Surprisingly, we found that most subpallial cholinergic neurons are derived from *Fgf8*^*+*^ RPC lineage progenitors. The *Fgf17*^*+*^ lineage generates half as many subpallial cholinergic neurons. Fgfs and/or other genes essential for RPC progenitor development likely impact cholinergic neurogenesis. Indeed, Fgf8 is essential for cholinergic development in the nucleus basalis of Meynert: Pombero *et al*. proposed that RPC Fgf8 attracts migration of cholinergic cells from pallial origins to the subpallium [36]. We did not see evidence for pallial-subpallial migration in our studies, although a small fraction of subpallial cholinergic cells may have pallial origins independent of *Fgf8/17*^*+*^ lineages. Alternatively, we propose that Fgf8 impacts cholinergic development through its effects on ventromedial patterning/survival [10] and the development of RPC-derived cells in the septum and vMGE.

It is interesting that the *Fgf8*^*CreER/+*^ and *Shh-Cre* [37] fate maps share several results. Both labeled a substantial fraction of the PV^+^ neurons of the globus pallidus, and both labeled PV^+^ and cholinergic interneurons of the striatum. *Fgf8*^*CreER/+*^ and *Fgf17*^*CreER/+*^experiments labeled cholinergic neurons in the septum, diagonal band, and nucleus basalis of Meynert; while it is likely that *Shh-Cre* may have also labeled these cells, that analysis was not performed. In any case, *Fgf8*^*CreER/+*^ descendants that populate the progenitor zone of the ventral MGE (see MGE domain 5 in Figure 5) most likely overlap with *Shh*^*+*^ progenitors in this region, explaining the commonalities in the fate maps.

### Stochastic developmental mechanisms may contribute to VZ heterogeneity

In *ROSA26R* fate maps, we observed mosaic βgal expression in the OB, cortex, septum, and MGE VZs. Tm-induced CreER activation results in mosaic recombination of floxed alleles; this technicality likely contributes to mosaicism in our fate maps. We posit that colocalization data for cell types with the highest contributions of Xgal^+^ cells (Tbr1^+^ DBB neurons, septal cells, basal ganglia cholinergic neurons) report the efficiency of CreER activation in our *Fgf8*^*CreER/+*^ experiments at 60% to 80%. (The *Fgf8*^*+*^ lineage may actually generate up to 100% of these cell types). Corollary to this, we hypothesize that mosaicism below this threshold in other structures reflects contributions from progenitors outside the *Fgf8*^*+*^ lineage. Indeed, our E10.5-E13.5 results indicate that Fgf lineage cells from the RPC become interspersed with unlabeled cells - that is, progenitors of other origins - in the VZ of the OB, cortex, and ganglionic eminences. This supports the conclusion that there is a true biological basis for VZ mosaicism in our fate maps. The intercalation of progenitors from diverse embryonic sources could be an important mechanism by which the telencephalic VZ becomes a genetically mosaic population. This model of heterogeneous VZ has been proposed and supported previously by studies of cortical projection neuron progenitors [38].

In the neocortex, *Fgf8*^*+*^*/17*^*+*^ lineage cells tended to appear in radial columns or larger blocks of cells spanning the width of the cortical plate (Figure 3, Additional file 3: Figure S3). Recent studies have provided evidence that ontogenetically related cortical neurons (within radial columns) are functionally related, make preferential connections with one another, and in some instances may be coupled by gap junction communication [39,40]. According to this model, *Fgf8*^*+*^*/17*^*+*^ lineage clones in the cortex are expected to serve as functional units, although we do not yet understand how they differ from neighboring non-*Fgf8*^*+*^*/17*^*+*^ lineage clones. Notably, we observed left-right asymmetries as well as individual variation in cortical fate maps. Although stochastic differences in Tm-induced CreER activity may contribute to these effects, we propose that our results likely also reflect authentic heterogeneities in the distribution of *Fgf8*^*+*^*/17*^*+*^ lineage progenitors. Previously, behavioral differences between genetically identical twins have been attributed to environmental, epigenetic, and rare genetic factors [41]. Stochastic variation in the distribution of telencephalic progenitor subtypes distribution may also contribute to individual differences. This would have broad implications for developmental biology and neuropsychiatry.

Additional studies are needed to elucidate mechanisms underlying the variable, asymmetric distribution of Fgf lineage cells in the telencephalon. We have not detected a left-right asymmetry of *Fgf8* mRNA expression in the early telencephalon. However, *Fgf8* mRNA has a striped and graded expression pattern in the RPC at E9.5 [18] that could result in subtle left/right differences. It is also possible that posttranscriptional regulatory mechanisms generate asymmetric protein expression in the RPC from bilaterally symmetric mRNA. Alternatively, *Fgf8* could elicit cellular responses in nearby neuroepithelium that secondarily contribute to asymmetry, as has been reported in other developmental contexts [42-44].

### Fgf8 and Fgf17 restrict the spread of RPC-derived cells within the telencephalon

Cre fate mapping in *Fgf8* and *Fgf17* mutant embryos revealed novel functions of these genes in RPC progenitor development: disruption of either gene resulted in aberrant progenitor number and distribution in the rostral telencephalon. This phenotype was most striking in *Fgf8* mutants. At this point, we do not understand the mechanisms underlying the tangential spread of the mutant cells between E8.5 and E10.5. However, our slice culture data suggest that this may reflect early differences in progenitor distribution as we did not detect movement of labeled cells after E10.5 (Figure 7). Fgf8 promotes progenitor proliferation and survival [10]; thus, reduced Fgf8 signaling may negatively impact the number of Fgf8-lineage cells. Future studies are needed to fully elucidate how Fgf signaling restricts the spread of RPC-derived cells.

## Conclusions

Our studies revealed that the RPC has at least two partially distinct progenitor subpopulations (*Fgf8*^*+*^ and *Fgf17*^*+*^) which contribute to both cortical and subcortical telencephalic neurons, and whose distribution/dispersion is restricted by Fgf signaling. Surprisingly, *Fgf8*^*+*^ and *Fgf17*^*+*^ progenitors generate most of the cholinergic neurons in the basal ganglia. The mosaic distribution of *Fgf8*^*+*^ and *Fgf17*^*+*^ progenitors in the telencephalon VZ suggests that they are interspersed with progenitor subtypes of different origins. This provides novel insight into a mechanism that may underlie regional growth and cellular complexity in the forebrain. To fully appreciate the significance of our findings, we need a greater understanding of how heterogeneous progenitor subtypes differentially contribute to forebrain development and function. Future studies using our CreER knock-in lines could begin to address these questions by labeling, isolating, and characterizing different telencephalon progenitor subpopulations.

## Methods

### Generation of knock-in mice

All animal care and procedures were ethically approved and performed according to the University of California at San Francisco Laboratory Animal Research Center IACUC guidelines, under the following protocol number: AN098262.

CreER^T2^ cDNA was provided by P. Chambon (IGBMC), and targeting vectors were generated using the pGKneoLox2DTA.2 vector (from P. Soriano, Mt. Sinai School of Medicine). Homology arms were PCR amplified from BAC templates (Fgf8, RP24-272H16; Fgf17, RP24-312 N2; CHORI). All PCR amplified sequences and junctions between genomic DNA and knocked in sequences were sequence verified.

Linearized targeting vectors were electroporated into PrxB6T ES cells. Correctly targeted neomycin-resistant ES cell clones were identified by Southern blot analysis (probes described in Supplementary materials; Additional file 6) and injected into blastocysts to generate chimeras. Germline transmission from chimeras gave rise to founders.

Knock-in mice are routinely genotyped by PCR from tail biopsy genomic DNA using the following primers: *Fgf8*^*CreER*^ (Fgf8.34: gtcgacgaaccagcaagtgcaacagcct, CreERT2.51: gagacggaccaaagccacttg), targeted band 568 bp; and *Fgf17*^*CreER*^ (Fgf17f1: gcctgctgcctaaccttacc, Fgf17r1: ccc tgtgtttgacagcagaga, CreERT2.51), wild type band 212 bp, targeted band 515 bp.

### Other mouse lines used

We used the following previously described mouse lines: *Fgf17*^*−*^ (D. Ornitz, Washington University, St. Louis; [45]), *Fgf8*^*neo*^ and *Fgf8*^*−*^ (G. Martin, UCSF; [33]), *β*-*actin:: Cre* [26], *ROSA26R* [27], *Tau*^*lox-STOP-lox-mGFP-IRES-NLS-LacZ-pA*^ (*TauR*; [28]), mT/mG [46].

### Tamoxifen administration and Xgal staining

In timed matings, noon on the day of vaginal plug was designated E0.5. Tamoxifen (Tm) was dissolved in corn oil and administered to pregnant dams by oral gavage at a dose of 75 mg Tm/kg body weight.

Whole mount embryos were fixed briefly on ice in FA/GA (2% formaldehyde, 0.2% glutaraldehyde, 1× PBS) and incubated overnight at 37°C in Xgal staining buffer (1 mg/mL Xgal, 5 mM potassium ferricyanide, 5 mM potassium ferrocyanide, 2 mM MgCl_2_, 0.01% sodium deoxycholate, 0.02% NP-40, 1× PBS). For cryosections, embryos and adult brains were fixed 1 to 2 h on ice in 4% PFA, incubated in 30% sucrose/PBS overnight, and frozen in OCT. After sectioning (E9.5-E10.5: 10 μm, older samples: 20 μm), slides were rinsed in PBS, fixed in FA/GA, incubated overnight at 37°C in Xgal staining buffer, rinsed in PBS, and mounted used Aquamount (Lerner Laboratories, Radnor, PA, USA).

All fate mapping experiments and *in situ* hybridizations were conducted with three or more embryos from a minimum of two independent, tamoxifen-treated litters, except for those otherwise indicated in Table 1. Representative examples are shown in the figures.

### Immunohistochemistry (IHC)

Cryosections were rinsed in PBS, blocked in 10% normal serum/PBST (1× PBS, 0.1% Triton X-100), incubated in primary antibody overnight (4°C), washed in PBST, incubated in secondary antibody 1 to 3 h (room temperature), and washed in PBS. For fluorescent detection, we used Alexa 488- and Alexa 594-conjugated secondary antibodies (Invitrogen, Carlsbad, CA, USA); images were captured using a Zeiss 510 LSM NLO Meta confocal laser scanning microscope (pinhole 1.8 μm; Zeiss, Oberkochen, Germany). For colorimetric detection, biotinylated secondary antibodies (Vector, Burlingame, CA, USA) were used with the ABC (Vector)/DAB detection method.

Several modifications were made for ChAT IHC. Antigen retrieval was achieved by incubating slides for 15 min (2.94 g/L trisodium citrate dehydrate, 0.05% Tween-20, pH 6.0) at 90°C. Blocking and antibody incubations were done in 1% BSA in PBST. Sections were incubated two days with primary antibody, and signal was amplified with biotinylated anti-goat (Vector) prior to fluorescent detection with streptavidin-594 (Invitrogen).

We used the following antibodies: βgal (gp510 from Dr. Tom Finger; Cappel/Millipore 559762; Millipore, Billerica, MA, USA), Ctip2 (Millipore 25B6), Tbr1 (Abcam #ab31940; Abcam, Cambridge, UK), calretinin (Millipore AB5054), calbindin (Swant 04109; Swant, Fribourg, Switzerland), Nkx2.1 (Santa Cruz sc-13040; Santa Cruz Biotech, Dallas, TX, USA), ChAT (Chemicon AB144P; Chemicon International, Billerica, MA, USA), parvalbumin (Millipore MAB1572), GABA (Sigma A2052; Sigma-Aldrich, St. Louis, MO, USA), GFP (Invitrogen A11122), somatostatin (Santa Cruz sc-7819).

### *In situ* hybridization (ISH)

Whole mount ISHs were performed according to the Cepko/Tabin lab protocol (http://genepath.med.harvard.edu/~cepko/protocol/ctlab/ish.ct.htm).

Section ISHs were performed using digoxigenin-labeled riboprobes as described previously [47] with modifications detailed in Supplementary materials (Additional file 6).

### Brain slice culture for live imaging

Brains were dissected in cold Hanks buffered saline solution (HBSS) and kept on ice until being embedded for coronal sectioning in 4% low melt agarose (1× PBS). Brains were sectioned by vibratome, transferred to XX membranes, and allowed to recover at 37°C in DMEM for 1 h. DMEM was replaced with neurobasal medium (supplemented with B-27, glucose, pen/strep, and glutamine) and cultured at 37°C. T0 demarks the beginning of neurobasal medium culture.

#### Highlights

• Fgf^+^ progenitors leave the RPC and contribute to rostral telencephalic structures.

• Fgf17 marks a subset of the Fgf8 lineage in the RPC.

• Fgf8 and Fgf17 impact the telencephalic distribution of RPC-derived progenitors.

## Additional files

Additional file 1: Figure S1.
*Fgf8* and *Fgf17* expression and CreER activity in the early RPC. Additional file 2: Figure S2.
*Fgf8* ^*CreER*^ and *Fgf17* ^*CreER*^ fate maps at E13.5. Additional file 3: Figure S3. Asymmetric and stochastic distribution of labeled neurons in *Fgf8* ^*CreER/+*^; *TauR* and *Fgf17* ^*CreER/+*^; *TauR* cortices. Additional file 4: Figure S4. Fgf8/17 lineage neurons in the OB, cortex, and septum. Additional file 5: Figure S5. Fgf8 lineage cells in the basal ganglia. Additional file 6.
**Supplemental data (legends to supplemental Figures S1 to S5); and supplemental methods).**

## Abbreviations

ANR: anterior neural ridge
AOA: anterior optic area
ChAT: choline acetyltransferase
CreER: Cre-estrogen receptor (fusion protein)
DBB: diagonal band of Broca
Fgf: fibroblast growth factor
GP: globus pallidus
IHC: immunohistochemistry
ISH: *in situ* hybridization
LGE: lateral ganglionic eminence
MGE: medial ganglionic eminence
OB: olfactory bulb
PFC: prefrontal cortex
POA: preoptic area
PV: parvalbumin
RPC: rostral patterning center
SS: somatostatin
SVZ: subventricular zone
Tm: tamoxifen
vMGE: ventral domain of the medial ganglionic eminence
VZ: ventricular zone
