# Supplementary material for: Fgf signaling controls the telencephalic distribution of Fgf-expressing progenitors generated in the rostral patterning center
Source: Neural Dev. 2015 Mar 31;10:8. doi: 10.1186/s13064-015-0037-7 (PMC4416298; doi:10.1186/s13064-015-0037-7)
Supplement: Additional file 6. — Supplemental data (legends to supplemental Figures S1 to S5); and supplemental methods). [file 13064_2015_37_MOESM6_ESM.pdf]

SUPPLEMENTAL DATA

Supplementary Figure S1. *Fgf8* and *Fgf17* expression and CreER activity in the early RPC. *(supplement to Figure 1)* (A, B, D, E) Whole mount and (G, I) section ISHs showing *Fgf8* versus *Fgf17* mRNA expression at 15 somites (15s; E9.0), 17-18s (E9.0), and E10.5. Fgf8 and Fgf17 are expressed in similar domains of the RPC by the 12s stage (E9.0; see Figure 1) and through the 18s stage. By E10.5, they have segregated so that Fgf8 is strongly expressed in the medial RPC whereas Fgf17 is expressed more broadly in a lateral domain (and may be excluded from the midline). (C, F) Whole mount (frontal view) and (H, J) section (horizontal) Xgal staining of E10.5 RPCs after Tx E8.5. Although Fgf8 lineage cells are concentrated in the RPC at this stage, some are observed outside the *Fgf8* expression domain (arrowheads in H). Fgf17 lineage cells appear restricted to the *Fgf17* expression domain. Arrowheads in J demark the lateral extent of Xgal staining, which mirrors the Fgf17 expression domain. These differences in Fgf8 versus Fgf17 lineage dispersion are also apparent in whole mount Xgal stained embryos (C, F).

Supplementary Figure S2. *Fgf8^{CreER}* and *Fgf17^{CreER}* fate maps at E13.5. (*supplement to Figures 2 and 3*) Tx was administered at E8.5 to ROSA26R embryos of the indicated Fgf genotypes, and E13.5 coronal sections were Xgal stained to visualize the fate maps.

Supplementary Figure S3. Asymmetric and stochastic distribution of labeled neurons in *Fgf8$^{CreER/+}$; TauR* and *Fgf17$^{CreER/+}$; TauR* cortices. (*supplement to Figure 2 and 4*) (A-D, A’-D’) Xgal stained sections show left-right asymmetric and stochastic distribution of labeled cells in E18.5 neocortices and cortical VZs of (A-B, A’-B’) *Fgf17$^{CreER/+}$* and (C-D, C’-D’) *Fgf8$^{CreER/+}$* forebrains. A-D and A’-D’ are sections from different individuals of the indicated genotypes. Note the variability among individuals (e.g. compare B vs. B’) and left-right asymmetric distribution of labeled cells. Asymmetric distribution of Fgf17 and Fgf8 lineage cells is still evident at P40, as shown by anti-βgal IHC on coronal sections from (E) *Fgf17$^{CreER/+}$; TauR* and (F) *Fgf8$^{CreER/+}$; TauR* animals. (In *Fgf17$^{CreER/+}$* animals, labeled cells are concentrated in the dorsomedial cortex and so we only show this region for the P40 experiment.) All animals were administered Tx at E8.5.

Supplementary Figure S4. Fgf8/17 lineage neurons in the OB, cortex, and septum. (*supplement to Table 1*). (A, B) Anti-βgal IHC labeling Fgf lineage neurons in coronal sections through OBs of P40 (A) *Fgf8*$^{CreER/+}$*; TauR* and (B) *Fgf17*$^{CreER/+}$*; TauR* mice. The accessory olfactory bulb, granule cell layer, mitral cell layer, and periglomerular layer all contain significant populations of Fgf8 lineage cells. In the perinatal OB, the *Fgf8*$^{+}$ lineage also includes many VZ and SVZ progenitors (see Figure 2). In contrast, the Fgf17 lineage contributes only scarce mitral, periglomerular, and accessory olfactory bulb cells. (C, D) Confocal images of coronal sections through E18.5 OBs of *Fgf8*$^{CreER/+}$*; TauR* embryos show co-localization of βgal with mitral cell markers (C) Ctip2 and (D) Tbr1. We also observed sparse Fgf8 lineage interneurons (somatostatin (SS)$^{+}$) in the external plexiform layer and the intrabulbar part of the anterior commissure (data not shown). Abbreviations: a, accessory olfactory bulb; aci, intrabulbar part of the anterior commissure; e, external plexiform layer; g, granule cell layer; m, mitral cell layer; pg, periglomerular cell layer.

Fgf8 lineage cells contributed more prominently to the neocortex than Fgf17 lineage cells. Several lines of evidence suggested that most neocortical derivatives of Fgf8/17^{+} RPC cells were projection neurons. In *ROSA26R* fate maps, there were very few Fgf8/Fgf17 lineage cells in the MGE VZ (origin of cortical interneurons), but numerous Fgf8 (fewer Fgf17) lineage clones extended radially from the VZ into the cortical plate, as expected for projection neurons (Figure 2M-N, Suppl. Figures S2A-B, S3A-B'). In addition, many Fgf8 and Fgf17 lineage cortical neurons co-labeled with projection neuron markers Ctip2 and Tbr1 at E18.5 (F, G, data not shown), but very few co-labeled with interneuron markers calbindin (CB), PV, or SS (E, H, I, data not shown). Arrowheads indicate co-labeling in F-I. See also Table 1.

(J-O) The Fgf8 lineage contributed the majority of each septal neuronal subtype examined in co-localization studies (see also Table 1). For most subtypes, the Fgf17 lineage co-labeled with ~30% as many cells as the Fgf8 lineage. The exception to this was the Ctip2^{+} subpopulation, of which ~70% were labeled by Fgf8^{CreER} and only ~10% by Fgf17^{CreER} (Table 1 and data not shown). In (J-M), the midline is at the right edge of each photograph, and the left edge of the photograph approaches the outer margin of the LS. (N) Bilateral clusters of SS^{+} cells near the periphery of the LS were predominantly βgal^{+}. (O) Most ChAT^{+} cells in the MS also co-label with βgal. The diagonal band of Broca (DBB) also received prominent contributions from Fgf8 and Fgf17 lineages, likely due to short-range ventral migration of RPC-derived cells. (P-S)

Fgf8 lineage DBB cells were superficial to the CTIP2$^+$ domain and included most Tbr1$^+$ and cholinergic (ChAT$^+$) cells, ~50% of calbindin$^+$ cells, and ~30% of Nkx2.1$^+$ cells (see also Table 1). Fgf17^{CreER} labeled roughly the same proportion of Nkx2.1$^+$ cells but lower proportions of other subtypes in the DBB (Table 1, data not shown).

All co-localization experiments in Suppl. Figures S4 and S5 were imaged using a confocal microscope, were conducted on the *TauR* background (Tm E8.5), and were done in parallel with *Fgf8*$^{CreER/+}$*; TauR* and *Fgf17*$^{CreER/+}$*; TauR* animals. Primary data is only shown for *Fgf8*$^{CreER/+}$ animals but data for both lines are reported in Table 1.

Supplementary Figure S5. Fgf8 lineage cells in the basal ganglia. (*supplement to Table 1*) Confocal images of co-labeling in (A-I) *Fgf8*$^{CreER/+}$*; TauR* and (J) *Fgf17*$^{CreER/+}$*; TauR* mice to characterize Fgf lineage cell types in the (A-D) striatum, (E-H) globus pallidus, and (I, J) Nucleus basalis of Meynert. Tx was administered at E8.5. The Fgf8 lineage gave rise to most basal ganglia cholinergic cells in the basal ganglia and also contributed significant subpopulations of several other cell types. In contrast, the sparse Fgf17 lineage cells in the striatum, nucleus basalis of Meynert, and ventral pallidum were strongly biased to cholinergic (ChAT$^+$) fates (J, data not shown). (A-D) In the striatum, the Fgf8 lineage generated subpopulations of medium spiny neurons (Ctip2$^+$; (Arlotta et al., 2008)), SS$^+$ and PV$^+$ interneurons. (E-H) In the gp, the few Fgf17 lineage neurons were mostly NPAS1$^+$, whereas the Fgf8 lineage included Nkx2.1$^+$, Ctip2$^+$, PV$^+$, and NPAS1$^+$ neurons. (I, J) The Fgf8 and Fgf17 lineages both generated cholinergic neurons in the nucleus basalis of Meynert.

SUPPLEMENTARY EXPERIMENTAL PROCEDURES

Genotyping PCR

The following primer sets were used for genotyping: *Fgf8*^{CreER} (Fgf8.34: gtcgacgaaccagcaagtgcaacagcct, CreERT2.51: gagacggaccaaagccacttg), targeted band 568bp; and *Fgf17*^{CreER} (Fgf17f1: gcctgctgcctaaccttacc, Fgf17r1: ccc tgtgtttgacagcagaga, CreERT2.51), wild type band 212bp, targeted band 515bp.

Southern blot probes

Southern blot probes were generated with the following (primers; templates): Fgf8 5' external (Fgf8upst.51 - ttggacactggagtccgtctac, Fgf8upst.31 – catcttgcaaatgcaaacacgg;

RP24-272H16); neo (RHneo.51 - cagtctggagcatgcgctttag, RHneo.31 – ctcgtcctgcagttcattcagg; pGKneoLox2DTA.2), Fgf8 3' external (Fgf8intr5.51 - gcagtaggccagaacaggcag, Fgf8ex6.31 – gtagttgaggaactcgaagcgc; RP24-272H16), Fgf17 5' external (H5.51 - ccttctaccaagtgagctgtg , H5.31 – gctagccagttctctccttacagacagg; RP24-312N2), Fgf17 internal (H8.51 - gctagcggaccagggcgctatgac, H8.31 – gtatctgtctccacgatgag; RP24-312N2), Fgf17 3' external(Fgf17dnst.52 - ctgaccccagtcctgtgatgta , Fgf17dnst.33 – gcatctgaagacagctacagtg; RP24-312N2).

Section in situ hybridization protocol modifications

Prior to acetylation, sections were incubated with proteinase K (1μg/ml) and post-fixed in 4% PFA. Slides were equilibrated in NTT prior to antibody incubation (overnight, 4° C, AP-antidigoxigenin (Roche)), and then washed in NTT 3 x 30 minutes at room temperature. They were then washed 3 times in NTTML (NTT + 50 mM $MgCl_2$, 2 mM Levamisole) and transferred to BM Purple (Roche) for colorimetric detection (dark, 37° C). Slides were rinsed in water to stop color reactions, then postfixed (4% PFA overnight), dehydrated, incubated briefly in Xylenes, and coverslipped using Permount. *Acetylation buffer:* 1.33% (v/v) triethanolamine, 0.065% HCl, 0.375% (v/v) acetic anhydride. *Riboprobe block/hybridization buffer:* 50% formamide, 5x SSC pH 4.5, 1% SDS, 50 μg/ml yeast tRNA, 50 μg/ml heparin. *Antibody blocking buffer (NTT):* 0.15M NaCl, 0.1M Tris pH 8.0, 0.1% Tween-20.
